# Diffuse alveolar damage, acute respiratory distress syndrome (ARDS), and non-cardiogenic pulmonary edema. Part 1: ARDS endotypes, including systemic inflammatory response syndrome and sepsis, with dog and cat examples

**DOI:** 10.1177/10406387261418009

**Published:** 2026-03-04

**Authors:** Hannah E. Wong, Joseph P. Boyle

**Affiliations:** Department of Veterinary Medicine, University of Cambridge, Cambridge, UK; Department of Veterinary Medicine, University of Cambridge, Cambridge, UK

**Keywords:** alveoli, endotype, histology, hyaline membrane, lung injury, pathogenesis, septicemia

## Abstract

Diffuse alveolar damage (DAD)—one histologic manifestation of severe acute interstitial lung injury—includes a subset of cases with the clinical diagnosis of acute respiratory distress syndrome (ARDS). ARDS and DAD both involve acute damage to endothelial and alveolar epithelial cells, resulting in pulmonary edema. DAD has well-defined histologic stages, including cell exudation and hyaline membranes, followed by type II pneumocyte hyperplasia. More severe lesions progress to chronic interstitial fibrosis. ARDS and DAD have diverse causes in humans and animals, yet historically were viewed as universal pathways of tissue dysfunction irrespective of cause. Molecular data have suggested that ARDS has heterogeneous signatures in epithelial, endothelial, and inflammatory cells that can characterize the specific pathogenesis of individual cases and therefore support targeted treatment. The signatures are grouped into endotypes classified according to the mechanism of primary damage. The proposed ARDS endotypes are epithelial injury, endothelial injury, angiopathy, systemic inflammatory response, and local inflammatory response. We present the pathogeneses that form the foundation of the ARDS endotypes, including evidence from dogs and cats. Specific canine and feline causes of ARDS can be assigned to an ARDS endotype. For some etiologies, multiple endotypes are applicable, highlighting the need for increased resolution of the underpinning evidence to best support the accurate application of ARDS endotypes to clinical cases.

The lung is composed of airways, alveolar airspaces, the interstitium, and the pleura. The interstitium incorporates pneumocytes and other cells of the alveolar walls, blood vessels, and supporting stroma, including interlobular septa where present in relevant species.^27^ Inflammation targeting the lung interstitium is classified as interstitial pneumonia.^27^ Diffuse alveolar damage (**DAD**) is a histologic pattern that describes a specific acute interstitial pneumonia, affecting humans and animals (**Table 1**). Contrary to the name, DAD may be patchy in distribution.^131^ The histologic appearance of DAD is similar between humans and veterinary species, and includes alveolar edema, neutrophilic infiltrates, hyaline membranes, and type II pneumocyte hyperplasia.^8,83,157^ Acute respiratory distress syndrome (**ARDS**) and veterinary ARDS (**vetARDS**) are clinical syndromes describing human and animal patients in acute hypoxemic respiratory failure with specific clinical features (Table 1). DAD and ARDS/vetARDS are not synonymous because the modalities of detection are independent; ARDS requires clinical parameters, and DAD requires lung histology.^26^ Furthermore, ARDS may occur without DAD,^26,82^ and DAD may occur without ARDS.^26^

**Table 1.** Definitions relating to acute lung injury.

Name	Definition
Diffuse alveolar damage (DAD)	The histologic pattern of endothelial and alveolar lining cell injury that leads to fluid and cellular exudation and (in some cases) progresses to extensive interstitial fibrosis.^83^
Acute respiratory distress syndrome (ARDS)	A clinically defined hypoxemic respiratory failure with severe impairment in gas exchange and lung mechanics, a high case fatality rate (specific parameters defined and refined elsewhere).^57,62,125^
Veterinary acute respiratory distress syndrome (vetARDS)/veterinary acute lung injury (vetALI)*	Clinically defined as an animal patient exhibiting at least 4, and ideally 5, of the following parameters:^161^ • Acute onset <72 h of tachypnea and dyspnea • Identifiable risk factors present • Evidence of leakage from lung capillaries with no evidence of increased lung capillary pressure • Evidence of ineffectual gas exchange • Evidence of diffuse lung inflammation (BALF cytology or biomarkers, or on molecular imaging PET).
Acute lung injury (ALI)	A clinically defined set of parameters similar to ARDS, but with less severe effects on blood oxygenation.^13^
Experimental ALI in animals	A combination of histologic and physiologic parameters. The presence of 3 of the 4 parameters, histologic evidence of tissue injury, non-cardiogenic pulmonary edema, inflammation, and physiologic dysfunction.^106^
Systemic inflammatory response syndrome (SIRS)	A clinical syndrome associated with a systemic inflammatory response that may have an infectious or non-infectious cause.^96^
Sepsis	Life-threatening organ dysfunction caused by a dysregulated host response to infection.^138^
Non-cardiogenic pulmonary edema (NCPE)	The abnormal fluid accumulation in the lung interstitium or alveolar spaces that is not caused by cardiogenic causes or fluid overload.^152^

* This definition does not differentiate between vetALI and vetARDS. Revised vetARDS and related definitions are expected to be published in 2026.

ARDS may develop from progression of other clinical syndromes, such as sepsis, acute lung injury (**ALI**) or veterinary ALI, systemic inflammatory response syndrome (**SIRS**), or non-cardiogenic pulmonary edema (**NCPE**; Table 1). SIRS, aspiration pneumonia, and sepsis were the most common causes of vetALI/vetARDS in dogs and cats presented to a U.S. Intensive Care Unit (ICU).^8^ Pneumonia and sepsis were the 2 leading causes of ARDS in human ICU patients.^127^ Experimental large mammal models of ARDS, predominantly canine and ovine, are used for human translational clinical research.^54,124,146^

Historically, ARDS and DAD were thought to be universal pathways of tissue dysfunction independent of the initiating cause. Experimental models have identified therapeutic targets that reduce the severity of ARDS; however, those potential therapies have often failed in clinical trials. In human cases of ARDS, reduced ventilator tidal volume, plateau pressure, and driving pressure have improved outcomes^1,3^; however, supportive treatment has been the mainstay.^23^ The lack of translational relevance of pre-clinical ARDS models to the clinics has re-focused research on clinical cases of ARDS and DAD. In humans, post hoc analysis of 6 cohorts of randomly controlled clinical trials separated ARDS cases into 2 *subphenotypes*—a P1 hypoinflammatory phenotype and a P2 hyperinflammatory phenotype.^17,24,25,43,55,132,140^ P2 human patients have higher levels of IL6, IL8, and sTNFR1 and experience a clinically more severe state of shock. These subphenotypes have also been recreated on analysis of bronchoalveolar lavage fluid.^129^ On the basis of a meta-analysis of clinical trials, an expert panel recommended specific treatments for patients with a hyperinflammatory subphenotype. These patients benefited most from anti-inflammatory simvastatin treatment, which has endothelial-stabilizing properties, higher positive end-expiratory pressure, and a liberal fluid strategy, compared with hypoinflammatory ARDS patients.^69^ These treatment recommendations underscore the importance of subphenotype-targeted therapy and the potential for precision medicine approaches to improve outcomes.

For veterinary species, ARDS subphenotypes have been re-created in a pre-clinical ovine experimental model with broadly similar features of hyperinflammatory and hypoinflammatory phenotypes.^112,160^ Subphenotypes of ARDS in dogs and cats have not been defined.

Variation in ARDS is now thought to occur on etiologic, physiologic, and biological levels.^18,127^ Although the classifications have an arbitrary basis, they link to clinical test and therapeutic outcomes, highlighting potentially treatable traits that may be modeled experimentally.^18^ Mining clinical information for pre-clinical biomarkers has been successful in sepsis research; specific biomarkers related to sepsis mortality in children were predictive of mortality in mice challenged with experimental sepsis.^166^ Understanding subphenotypes of heterogeneous conditions may improve the analysis of pre-clinical models and, ultimately, the development of new therapeutic strategies. With that aim, the European Respiratory Society chose a research target to further divide ARDS subphenotypes into *endotypes*, producing discriminating groups based on multidimensional clinically measurable traits with a distinct pathophysiology.^69^

The pursuit of ARDS endotypes is facilitated by advances in molecular techniques, such as single-cell multi-omics and spatial transcriptomics. These tools have revealed that, even with similar clinical manifestations, different causes of ARDS induce heterogeneous cellular and molecular signatures in epithelial, endothelial, and inflammatory cells.^103^ Endotypes classified by mechanism of primary damage have been proposed, grouped into epithelial injury, endothelial injury, angiopathy, systemic host response, and local host response.^18^

For veterinary patients, the aim of subclassification of ARDS cases into endotypes is to lay the groundwork for developing targeted treatment trials and point-of-care testing. Veterinary diagnosticians working in pathology or interfacing fields can contribute to the creation and analysis of these data. Developing our understanding of the lesions initiated by ARDS endotypes will contribute to that goal. After reading this review, readers should be able to:

- Describe the histologic features of the classical phases of DAD.
- Differentiate the subphenotypes and endotypes of ARDS.
- Describe the classification of ARDS endotypes using examples from canine and feline pathology.

## The histologic features of the phases of DAD

**Figure 1.**
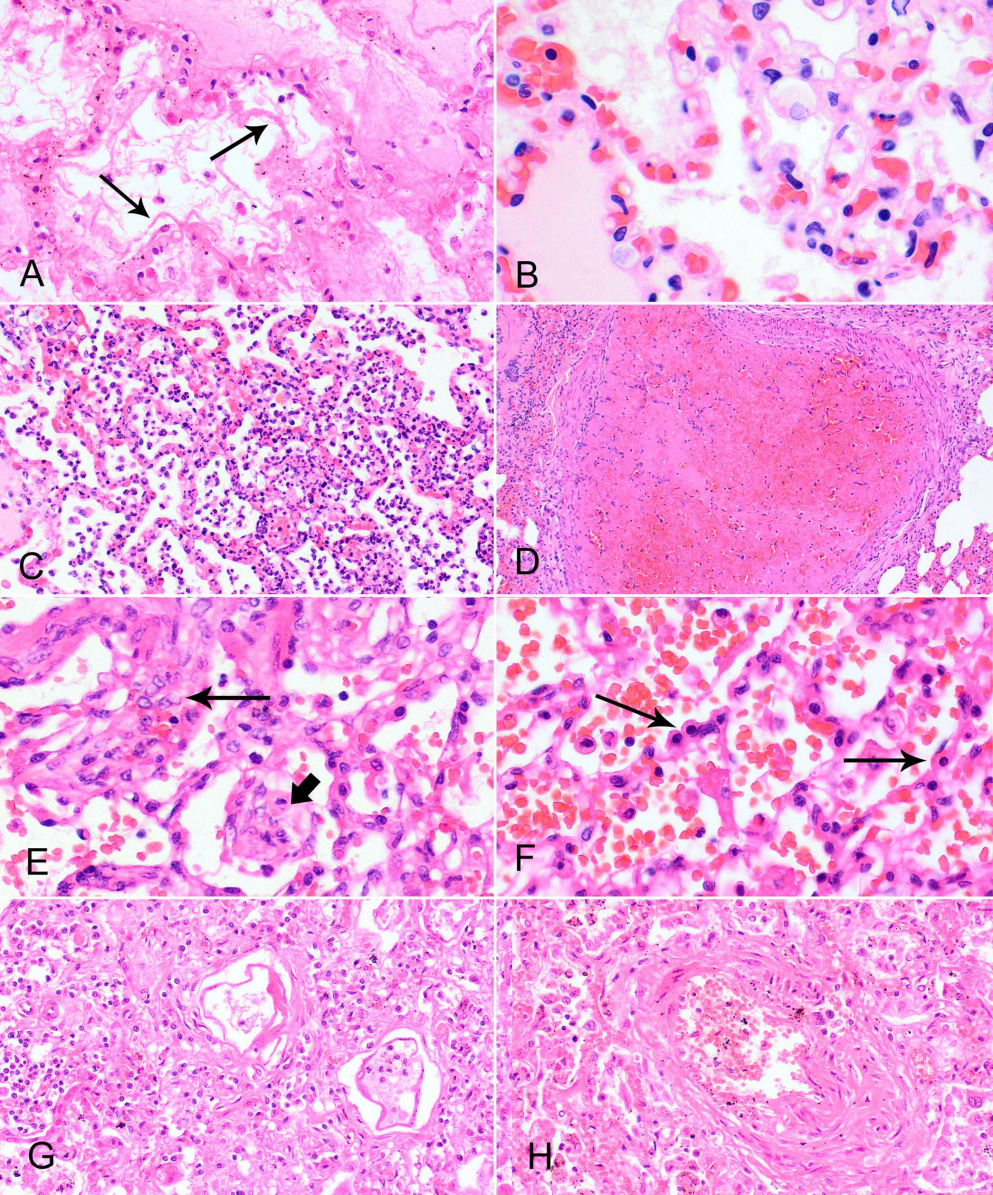
Classical histologic features of diffuse alveolar damage in dogs and cats. H&E. **A.** Hyaline membranes (arrows) in alveolar spaces, secondary to pulmonary hypertension associated with a congenital heart defect in a dog. **B.** Edema of alveolar septa, secondary to pulmonary thrombosis associated with renal amyloidosis in a dog. **C.** Neutrophil infiltrates in alveolar septa and alveolar spaces, acute interstitial pneumonia with concurrent abdominal necrotizing lymphadenitis, suspected to be caused by feline infectious peritonitis in a cat. **D.** Pulmonary thrombus with concurrent intestinal lymphoma in a dog. **E.** Interstitial (long arrow) and airway (short arrow) fibroblast proliferation; inhaled foreign body in a dog. **F.** Type II pneumocyte hyperplasia (arrows); inhaled foreign body in a dog. **G.** Chronic interstitial fibrosis with loss of air space and infiltration of mixed inflammatory cells; chronic pulmonary hypertension in a dog. **H.** Vascular remodeling as a result of chronic pulmonary hypertension in a dog.

The phases of DAD occur within a defined sequence (**Fig. 1**; **Table 2**).^27^ The initial injury initiates an exudative phase (Fig. 1A–D), which may last 0–7 d; followed by a proliferative phase of repair (Fig. 1E, 1F), commencing 3–7 d after the injury.^83^ Depending on the severity of the injury, the lesion may resolve after the proliferative phase; or, if more extensive or severe, it will progress to the fibrosing stage, with fibrosis becoming extensive at 14 d post injury (Fig. 1G, 1H).^83^

**Table 2.** Stages and key histologic features of diffuse alveolar damage.

Stage	Key histologic features
Exudative (acute)	Hyaline membranes along alveolar septa (Fig. 1A)
	Damage to alveolar septa, observed as interstitial and intra-alveolar edema ± hemorrhage (Fig. 1B)
	± Neutrophilic infiltrates (Fig. 1C)
	± Thrombosis of pulmonary vessels (Fig. 1D)
Proliferative/organizing (subacute)	Type II pneumocytes undergo hyperplasia (Fig. 1F)
	Type II pneumocytes secrete new basement membrane, if required
	Mononuclear cells infiltrate in the interstitium
	Fibroblasts and myofibroblasts proliferate in the interstitium, with rare focal involvement of airspaces (Fig. 1E)
Fibrosing (chronic)	Extensive fibrosis of alveolar septa or intra-alveolar spaces with proliferation of fibroblasts and myofibroblasts (Fig. 1G)
	Persistence of type II pneumocytes
	± Squamous metaplasia of airway or alveolar epithelia
	Vascular remodeling in areas of thrombosis (Fig. 1H)

### Exudative (acute) stage

Hyaline membranes are an essential feature of DAD.^26,27^ However, if the damage is <24 h old, histologic changes may be minimal. Furthermore, regardless of time post injury, hyaline membranes in experimental animal models of acute lung damage are variable; therefore, diagnosing experimental acute lung injury in the absence of hyaline membranes is possible but requires supporting physiologic data (Table 1).^106^ In veterinary diagnostic cases, histology suggesting acute lung damage—particularly in the absence of hyaline membranes—requires integration with the clinical context to achieve a diagnosis. This multidisciplinary approach is essential because of the nonspecific nature of the component histologic features.

### Proliferative or organizing (subacute) stage

Type II pneumocyte hyperplasia is an increase in cell number with concurrent hypertrophy. The hyperplastic type II pneumocytes may also demonstrate cellular atypia,^27^ including cytomegaly, karyomegaly, pleomorphism, and multinucleation.^165^ Type II pneumocyte hyperplasia occurs initially at 2–3 d post insult, becoming extensive at 7 d post insult.^83^ Individualized hypertrophied type II pneumocytes mimic reactive macrophages and may only be distinguishable by cytokeratin immunohistochemistry (IHC).^29^ TGFβ produced by injured type II pneumocytes and macrophages initiates myofibroblast and fibroblast activation and collagen deposition.^58^

### Fibrosing (chronic) stage

Fibroblasts and myofibroblasts proliferate within alveolar septa and infiltrate intra-alveolar fibrin, thereby generating extensive granulation tissue and contributing to fibrotic lung disease.^27^ The fibrosed alveolar exudates eventually get epithelialized and remodeled into the interstitium. Paracrine signaling from interstitial fibroblasts and alveolar macrophages can prevent type II pneumocytes from differentiating into type I pneumocytes.^156^ The production of TGFβ from alveolar macrophages and tissue factor from injured endothelial cells induces the recruitment of fibroblasts to the alveolar space, followed by deposition of collagen.^58,153^ Depending on the extent of damage and whether injury is ongoing, collagen deposition may contribute to repair of the alveolar barrier or may lead to extensive fibrosis, resulting in a permanent decrease in lung function.

## Pathophysiology of ARDS endotypes

**Figure 2.**
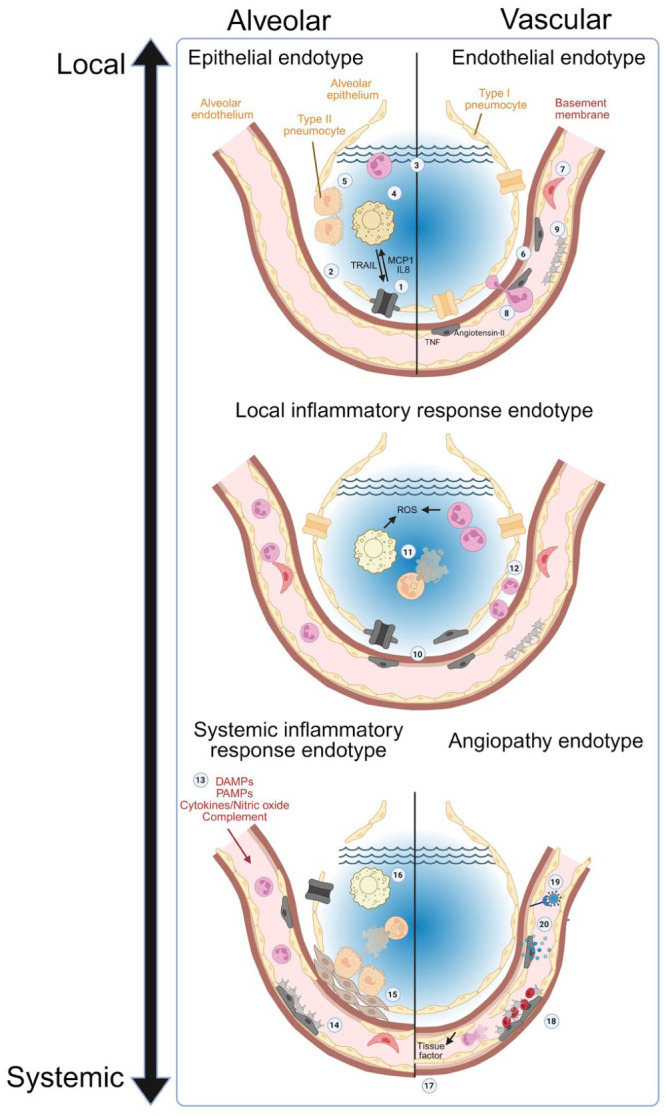
Hallmark aspects of distinct acute respiratory distress syndrome endotypes. **Epithelial endotype: 1.** Physiologically, tight junctions between epithelial cells restrict fluid movement, and alveolar fluid is removed by water channels on pneumocytes. Pneumocyte damage decreases water removal from the alveolar space and reduces expression of junctional proteins. **2.** Pneumocyte death leads to incomplete barrier function at the alveolar epithelium. **3.** Alveolar edema accumulates. **4.** Alveolar macrophages are activated by damaged pneumocytes releasing MCP1 and IL8. The activated macrophages then release TNF-related apoptosis-inducing ligand (TRAIL). **5.** Type II pneumocytes undergo hyperplasia to repair the epithelial barrier. **Endothelial endotype: 6.** Damaged and dying endothelial cells expose the basement membrane, initiating coagulation. **7.** Depending on species and previous damage, pulmonary intravascular macrophages (PIMs) may become active. **8.** Expression of adhesion molecules and release of chemokines results in neutrophil sequestration. **9.** Prothrombotic factors promote platelet activation. **Local inflammatory response endotype: (1–9)** features both epithelial and endothelial endotypes. **10.** Damage to the epithelial and endothelial layers exacerbates both inflammation and tissue dysfunction. **11.** Activated macrophages and neutrophils accumulate in the alveolus, contributing to tissue damage through reactive oxygen species (ROS) generation and neutrophil NETosis. **12.** Activated leukocytes infiltrate the interstitium further disrupting gas exchange. **Systemic inflammatory response endotype: 13.** Circulatory inflammatory mediators and immunostimulatory molecules (PAMPS/DAMPS/cytokines and activated complement) arrive from remote inflammation and can cause endothelial cell apoptosis. **14.** Extensive regions of the endothelium are in a pro-thrombotic state. Induction of coagulation leads to clotting and potential dead space generation. **15.** When damaged epithelial cells are not replaced efficiently, fibroblasts are recruited and fibrosis occurs. **16.** Efferocytosis of neutrophils by alveolar macrophages is suppressed, leading to bystander tissue damage. **Angiopathy endotype: 17.** Vasoconstriction reduces blood flow and increases blood pressure. **18.** Platelets, RBCs, and other insoluble blood components are deposited on endothelial cells, forming microthrombi. **19.** SARS-CoV-2 induces the angiopathy endotype. **20.** Further endothelial damage is induced by cytokines released by damaged endothelial cells.

The aim for both human and veterinary medicine is to use the ARDS endotypes to improve diagnostic and therapeutic precision in the treatment of ARDS. The premise of the ARDS endotypes is that original injury confers a retained molecular signature to the ARDS process that can be differentiated from other types of primary injury. Based on the foundation of the ARDS subphenotypes in people, ARDS etiologies were classified into 5 prospective endotypes (**Fig. 2**),^18^ noted below, but the molecular signatures of these endotypes remain a current area of research.

1. Epithelial—alveolar epithelial cells are injured, impairing fluid clearance.
2. Endothelial—vascular barrier disruption.
3. Local inflammatory response—focal airway or interstitial lung damage propagate to diffuse alveolar injury.
4. Angiopathy—multi-organ or systemic pro-thrombotic endothelial phenotype leads to microvascular injury.
5. Systemic inflammatory response—ARDS develops secondary to systemic vascular dysregulation induced by systemic insults, such as sepsis or pancreatitis.

Determining the unique features of different endotypes is challenging because of the close spatial and functional relationships between the lung epithelial and endothelial cells, which are closely opposed on either side of a fused basement membrane to optimize gas exchange.^79^ Alveolar edema can be initiated through endothelial or epithelial changes. Endothelial activation and damage increases vascular permeability.^114^ Alveolar epithelial damage decreases alveolar fluid clearance through a loss of ion transporters and disruption to impermeable tight junctions.^105,107^ To further complicate the distinction between endotypes, primary damage in one compartment can induce inflammation in another compartment by releasing danger-associated molecular patterns (**DAMPs**), secreting cytokines sensed by other cell types, or causing off-target immune-induced lesions.^77^ An example includes early-phase COVID pneumonia in people, in which COVID-19–infected alveolar epithelial cells produce the transcription factor STAT3 that induces hyperexpression of indoleamine-2,3-dioxygenase 1 (IDO) by endothelial cells. This results in pulmonary vessel dilation, diffusion-perfusion mismatch, and local tissue hypoxia.^32^ Another example is the expression of E-selectin by activated lung endothelial cells. This facilitates neutrophil infiltration into the pulmonary interstitium,^151^ contributing to secondary necrosis of alveolar epithelial cells.^99^ Multi-layered cross-talk also explains why DAD, caused by heterogeneous processes, quickly progresses to morphologic homogeneity. For each ARDS endotype, we explore the potential mechanisms contributing to the early injury responsible for initiating the ARDS process. We then discuss veterinary conditions that may result in ARDS and explore how the ARDS endotypes may be applied to those examples (**Table 3**; Fig. 2).

**Table 3.** Acute respiratory distress syndrome (ARDS) endotypes with clinical examples from cats and dogs.

ARDS endotype	Pathophysiology	Example	Features in DAD
Epithelial	Primary injury targets alveolar epithelial cells	Feline calicivirus Canine influenza Ventilator injury Surfactant issues	Pneumocyte necrosis Alveolar edema URT and airway epithelial may be affected
Endothelial	Primary injury targets lung endothelial cells	Uremic pneumonitis	Reactive endothelial cells Interstitial and alveolar edema Neutrophil sequestration in vessels Thrombosis
Local inflammatory response	The inflammatory focus is within the lung and includes nonspecific damage to multiple cell types	Extension of bronchopneumonia*	Indiscriminate cell damage, affecting multiple anatomic compartments
Angiopathy	Vascular dysregulation or lesions result in lung microthrombi in the absence of primary extravascular inflammation	Not applicable, see text for discussion	Microthrombi Vessel fibrinoid necrosis
Systemic inflammatory response	An extra-pulmonary inflammatory focus caused systemic activation of inflammation	Sepsis Pancreatitis	Pulmonary edema Sequestration of neutrophils in lung Lack of airway involvement

DAD = diffuse alveolar damage.

* As discussed in the text, bronchopneumonia may also cause a systemic inflammatory response ARDS endotype by inducing sepsis.

### Epithelial endotype

In the epithelial endotype, the primary insult damages alveolar epithelial cells. Both type I and type II pneumocytes play roles in the removal of alveolar fluid, via expression of ion transporters and water channels.^128^ Paracrine signaling—for example, by catecholamines—can increase alveolar fluid clearance. Cell injury results in down-regulation or loss of transporters and decreased expression of tight and adherens junctional proteins, resulting in alveolar edema.^65,107^ Type I pneumocytes are very sensitive to injury, including biomechanical factors such as alveolar stretch.^108^ Activation of alveolar macrophages induces release of TNF-related apoptosis-inducing ligand (TRAIL), which results in the paracrine-initiated apoptosis of alveolar epithelial cells, further potentiating the primary damage.^73^ Damaged alveolar epithelial cells release cellular proteins that, alongside surfactant and plasma proteins, contribute to hyaline membrane formation.^147^ Activated alveolar epithelial cells also recruit leukocytes independently of alveolar macrophage signaling through the release of the chemokine monocyte chemoattractant protein 1 (MCP1) and IL8.^150^

### Endothelial endotype

In the endothelial endotype, processes cause primary damage to lung vascular endothelial cells, resulting in dysregulation of lung function that ultimately progresses to ARDS. Endothelial cells have multiple key functions in lung homeostasis that may be dysregulated in ARDS and DAD. These functions include water transport out of vessels and production of inflammatory and vasoactive compounds.^101,111^ Endothelial cells respond to injury and inflammation by transforming from an anti-adhesive, low-permeability barrier to an adhesive, high-permeability layer. This transformation is achieved through expression of adhesion molecules and pro-thrombotic activators, and response to (and paracrine production of) vascular and inflammatory mediators, such as TNF, IL8, and angiotensin II. Heterogeneity in the responses from spatially distinct areas in the lung is influenced by the local microenvironment,^101^ potentially contributing to the multifocal distribution of tissue injury in DAD.

Endothelial damage exposes the basement membrane and initiates coagulation, facilitating the development of ARDS and DAD, because activated platelets are effective activators of neutrophils.^21^ Inhibition of platelet-derived neutrophil activation in an experimental mouse model of acute lung injury reduced neutrophil recruitment, lung permeability, and improved survival.^170^ Mechanical factors also increase the sequestration of activated neutrophils in alveolar capillaries. Activated neutrophils undergo cytoskeletal re-arrangements that make them more resistant to deformation when traveling through alveolar capillaries that are narrower than a single neutrophil.^22,47^ The loss of elasticity—combined with changes in expression of endothelial adhesion molecules—facilitates cross-talk between endothelial cells and neutrophils, priming neutrophils to emigrate into the lung tissue.^22^

### Local inflammatory response endotype

Lung inflammation that extends locally may damage the pulmonary interstitium; and if severe, it may induce ARDS and/or DAD. Induction of ARDS and DAD in this way is predominantly secondary to airway inflammation. Inflammation in airspaces may directly or indirectly damage epithelial cells—for example, when neutrophilic exudates release reactive oxygen species (ROSs) or activate alveolar macrophages. Alveolar macrophages are central modulators of alveolar inflammation by controlling immune responses and epithelial barrier permeability, and by influencing apoptosis or necrosis of alveolar epithelial cells.^119^

### Angiopathy ARDS endotype

In the angiopathy endotype, endothelial dysfunction results in vasoconstriction and a procoagulant state, potentially inducing microthrombi and causing organ ischemia. The ARDS angiopathy subgroup primarily reflects the angiopathy diagnosed by imaging in human patients with COVID-19.^30,118^ In these patients, SARS-CoV-2–infected endothelial cells result in lung angiopathy and microthrombosis.^154^ Interactions between complement and tissue factor–enriched neutrophil extracellular traps (**NETs**) is one mechanism by which SARS-CoV-2 induces thrombotic microangiopathy.^141^ The Syrian golden hamster model of SARS-CoV-2 infection recapitulates the microangiopathy with primary endothelial damage, subsequent platelet aggregation, and perivascular and subendothelial macrophage infiltration.^9^ Felids are susceptible to natural and experimental infection with SARS-CoV-2, whereas dogs are relatively resistant.^134^ SARS-CoV-2 naturally infected domestic cats or nondomestic felids did not have pulmonary microthrombotic changes at postmortem examination.^53,117^

Outside of SARS-CoV-2 models, angiopathies as a primary cause of vetARDS are less well-recognized, partly because most primary veterinary vasculopathies could be classified as an endothelial endotype. Potentially relevant angiopathies for this endotype could include disseminated intravascular coagulation, progressing to ARDS not associated with sepsis or pneumonia.^42,45^ Severe cases of babesiosis occasionally result in ARDS,^37,45,95^ and some causes of NCPE that progress to ARDS may be relevant to this endotype.^165^ Cases of canine cutaneous and renal glomerular vasculopathy have not been associated with substantial lung involvement.^74^ Considering the low incidence of angiopathies not otherwise attributed to primary endothelial injury in veterinary medicine, we suggest it may be more appropriate to consider the angiopathy vetARDS endotype as a subset of the endothelial endotype, rather than a distinct group.

Clotting dysfunction is a known component of ARDS. Consistent differences in coagulation parameters are present between the P1 hypoinflammatory and P2 hyperinflammatory ARDS subphenotypes in humans, with P2 patients having elevated procoagulant and antifibrinolytic markers.^98^ However, in ARDS, complement activation exacerbates inflammation induced by other primary triggers—rather than complement dysfunction being the primary disease.^7^ In veterinary medicine, this is corroborated by the finding that primary coagulopathies are not considered a risk factor for canine vetARDS.^42^

### Systemic inflammatory response endotype

In ARDS developing from sepsis or SIRS, a pre-existing systemic inflammatory response and endothelial activation are present. The pathogenesis of the endothelial endotype also applies here and because of widespread endothelial activation, septic-ARDS cases are at high risk of systemic circulatory dysfunction. Sepsis and SIRS also amplify inflammation through the neuroendocrine-immune network, supporting further widespread homeostatic dysfunction and inflammation. Septic-ARDS and SIRS-ARDS include endothelial injury, pulmonary edema, cell death, oxidative stress, pulmonary microcirculation dysfunction, cytokine release, inflammatory signaling pathways, complement activation, and coagulation.^109,169^ Some of these factors are explored in more detail in Part 2, where we describe the subsequent cellular processes that follow the primary damage.^165^

### Endothelial injury and pulmonary edema

The key feature in the progression of sepsis to septic ARDS is the sustained increase in pulmonary vascular permeability.^169^ Single-cell RNA sequencing of peripheral blood macrophages from human sepsis patients with and without ARDS had different expression patterns. ARDS patients had significantly upregulated genes related to endothelial barrier disruption, interferon responses, and fibrosis and genes that block neutrophil clearance.^80^ Sheep exposed to bacterial pneumonia–induced sepsis progressed to ARDS with pulmonary vascular hyperpermeability caused by vascular endothelial growth factor (**VEGF**) overexpression.^93^ In a mouse model of ALI-sepsis, expression of angiogenic genes *Ang1*, *Tie2*, and *Vegfr2* was reduced in the lung, kidney, and liver, which was associated with increasing capillary permeability and systemic dysregulation of vascular endothelial integrity.^15^

### Cell death and oxidative stress

Cell-death pathways are activated during sepsis and SIRS. In a mouse model of sepsis-induced lung injury, caspase-dependent apoptosis and pyroptosis of pulmonary microvascular endothelial cells contributed to tissue injury.^31,67^ Conversely, sepsis extends the lifespan of neutrophils through suppression of neutrophil cell death and debris clearance pathways. Human cases of septic shock and sepsis-ARDS have significantly lower rates of neutrophil apoptosis compared with uncomplicated sepsis, non-septic ventilated patients (including ARDS patients), and healthy controls.^59^ The finding has been recapitulated in ARDS patients, where genes that block neutrophil clearance were upregulated.^80^

During sepsis and SIRS, redox homeostasis is disrupted, resulting in oxidative stress. ROSs produced by NADPH oxidase complexes in neutrophils are a major contributor to the disruption.^33,121^ Oxidative stress induces a prothrombotic state in lung endothelial cells, while simultaneously impairing vasodilation, increasing capillary permeability, and increasing leukocyte and platelet endothelial adhesion.^81^ These factors increase the likelihood of lung capillary endothelial injury.

### Microvascular dysfunction

Lung microvascular abnormalities occur during sepsis and are caused by a range of processes, including endothelial dysfunction, glycocalyx alterations, WBC and platelet adhesion, and changes to RBC deformity.^39^ The mechanical influences on activated neutrophils already described in the endothelial endotype section also apply to the angiopathy endotype. Together, these changes support the aggregation of neutrophils^116^ and RBCs^120^ in the pulmonary circulation, contributing to vascular inflammation, thrombosis, and dead-space generation. Dead space further potentiates microvascular dysfunction because hypoxic environments cause neutrophil degranulation and increase neutrophil-mediated injury in the lung.^71^

### Inflammation and cytokine release

Activation of classical proinflammatory innate immune signaling pathways, such as NF-κB, JAK2/STAT3, MAPK, mTOR, and Notch, in pulmonary-resident or infiltrating cell populations support the transition of sepsis into sepsis-associated ARDS.^97^ Many cytokines have roles in septic-ARDS, including IL1β, IL18, IL6, IL12, IL17, and TNF.^169^ Levels of IL1β, IL6, IL8, IL12, IFNγ, GMCSF, and TNF were higher in human non-survivors of sepsis compared with survivors, with IL8 levels predictive of a fatal outcome in multivariate analysis.^110^ The association between IL18 elevation and clinical severity is evidenced by higher IL18 levels in human sepsis cases compared with SIRS; levels also are significantly higher in septic-ARDS than in sepsis alone.^48^ In a randomized controlled clinical trial in human medicine, elevated IL18 levels were associated with mortality in sepsis-induced ARDS.^126^ Circulating proinflammatory cytokines impact lung permeability by directly influencing transcription of genes that help control endothelial permeability (e.g., IL1β can suppress transcription of VE-cadherin in lung endothelial cells, contributing to sepsis-induced lung injury).^168^

### Pulmonary intravascular macrophages, complement and coagulation

Pulmonary intravascular macrophages (**PIMs**) contributed to neutrophil aggregation in the lungs of calves challenged with an intravascular pathogen.^139^ PIM-mediated neutrophil aggregation in response to intravascular pathogens also has been suggested to occur in other species in which constitutive PIMs (such as the cat^20^) or induced PIMs (such as the dog^155^) are present.^130^ We discuss PIMs in more detail in part 2 of our review.^165^

Innate immune responses in sepsis also activate complement that results in thromboinflammation.^102^ A cohort of human sepsis cases had increased levels of circulating cleaved complement protein C3a and higher C3a/C3 ratios, compared with both healthy controls and cases of SIRS without an identified inflammatory focus.^145^ ARDS does not appear to induce levels of complement activation beyond that induced by sepsis, given that C3a levels and the C3a/C3 ratio did not differ between sepsis cases who developed ARDS and those who did not.^145^ Vascular-related biomarkers (generated from canine patient data) for canine sepsis and SIRS have been proposed; higher levels of angiopoietin-2 at hospital admission was correlated with a negative outcome in both SIRS and sepsis, and VEGF was elevated in dogs with sepsis compared with healthy dogs.^89^

### Immune dysregulation

SIRS and sepsis are processes of immune dysregulation, given that hyperinflammation occurs contemporaneously with a compensatory anti-inflammatory response syndrome (**CARS**).^138^ The balance between the 2 responses can result in periods of hyperinflammation or immune paralysis, ultimately modifying the clinical features of individual cases. This multi-directional immune dysregulation is captured in the overarching term—persistent inflammation, immunosuppression, and catabolism syndrome (**PICS**).^64^ In a cohort study of human pneumonia-induced sepsis, in which 583 patients developed severe sepsis and 149 died, the greatest risk of death was associated with high levels of both proinflammatory IL6 and anti-inflammatory IL10.^84^ On a cellular level, immune paralysis manifests as activation of regulatory immune cells, lymphocyte exhaustion, and abnormal immune cell death.^115^ Lung tissue from human patients dying of sepsis contained expanded populations of immunosuppressive cells, and lung epithelial cells had increased expression of the inhibitory receptor ligand PD-L1.^16^

## Specific examples of causes of vetARDS in dogs and cats classified by ARDS endotype

### Cytolytic respiratory viruses—epithelial ARDS endotype

Influenza A virus preferentially infects respiratory epithelia in dogs^28,35^ and contributes to the canine infectious disease complex.^38^ Infection in the cat is often subclinical.^135^ For both dogs and cats, influenza can progress to clinical signs consistent with ARDS,^35,143^ but this requires specific strains and relatively high exposure doses. In dogs, highly pathogenic influenza A(H5N1) virus induces clinical signs consistent with ARDS and acute neutrophilic interstitial pneumonia with fibrin exudation^35,143^; in cats, it causes confirmed DAD with clinical signs consistent with ARDS.^86^ In these cases, virus was detected using IHC within alveolar epithelial cells and luminal leukocytes, suggesting that viral-induced epithelial damage was a factor in the lung damage.

Feline calicivirus—an RNA virus that is part of the feline respiratory disease complex—causes rhinitis, pneumonia, and oral ulcers, with high morbidity and low mortality in immunocompetent cats. Feline calicivirus can induce fatal acute pneumonia with pneumocyte necrosis, marked fibrin exudation, alveolar infiltrates of macrophages and neutrophils.^142^ These cases are speculated to have developed ARDS based on the fatal severity and acute nature of the histologic changes. Hyaline membranes were not described; hence, DAD was not confirmed. Viral antigen, detected by IHC, was present within pneumocytes and macrophages within alveolar spaces, supporting an ARDS epithelial endotype.

### Ventilator-induced lung injury and surfactant disorders—epithelial ARDS endotype

Ventilator injury and surfactant disorders are both classified as inducing epithelial endotype ARDS, ultimately through mechanical damage to pneumocytes. Ventilator injury can cause ARDS^158^ by overstretching of alveoli,^136^ or through sheer forces induced by repetitive collapse and re-expansion,^50^ both of which result in pneumocyte damage. Mechanical ventilation also induces surfactant dysfunction, similar to that caused by congenital surfactant disorders, which increases surface tension on pneumocytes and further increases sheer forces generated through breathing cycles.^2^ Presumed ARDS with confirmed DAD caused by a congenital surfactant disorder was identified in a family of Airedale Terriers.^46^

### Inhaled toxic gases and metabolized hematogenous agents—epithelial ARDS endotype

Inhalation of toxic gases and hematogenous agents, which are metabolized to forms that are toxic to pneumocytes, are reported causes of ARDS in veterinary species.^27^ The primary target is lung epithelial cells; thus, this process is classified as an epithelial ARDS endotype. For dogs and cats, toxic gases include 100% oxygen,^6^ nitrogen dioxide^70^ and chlorine.^72^ Paraquat exposure in dogs is an example of a hematogenous toxin metabolized by pneumocytes.^36,162^

For domestic animals, the source of many toxic gases is smoke inhalation,^60,144^ which is a documented cause of suspected ARDS in cats^51^ and dogs.^52^ Smoke inhalation may also cause thermal inhalation trauma to respiratory epithelia, although because of rapid cooling of air and smoke once in the upper respiratory tract, thermal burns are more often seen in cases of steam inhalation.^164^

### Aspiration pneumonia—epithelial and local inflammatory response ARDS endotype

*Aspiration pneumonitis* in human medicine is acute lung injury after the inhalation of regurgitated gastric contents, whereas *aspiration pneumonia* is the aspiration of colonized oropharyngeal material.^104,137^ In veterinary medicine, a strict distinction of those terms does not exist, and aspiration pneumonia can refer to both aspiration of sterile gastric contents and colonized oropharyngeal/gastric material.^133^ In a study of 46 client-owned dogs admitted to a US veterinary hospital, aspiration pneumonia was the most common risk factor (42%) for developing vetARDS or an autopsy diagnosis of DAD.^14^ Here, we describe the pathogenesis of sterile-acid aspiration and colonized material aspiration as 2 separate but related processes.

### Acid aspiration

Gastric acid aspiration results in damage to epithelium, followed by neutrophilic inflammation, loss of pulmonary microvascular integrity, and pulmonary edema.^122^ The volume of the aspirate contributes to the severity of the lesion.^123^ At very low volumes, when microinjected directly into rodent alveoli, acid causes pore formation in epithelial cells and hydrogen peroxide release, subsequently increasing ROSs in the perialveolar microvascular endothelium.^159^ A second study demonstrated primary acid-induced epithelial injury—without endothelial injury—using live confocal microscopy in an experimental mouse model of acid alveolar microinjection; however, it similarly found that epithelium-derived hydrogen peroxide induced endothelial cell retraction and barrier failure, resulting in pulmonary edema.^75^ In an experimental rat model, the pH and the volume of aspirated content influenced mortality. Low pH fluid caused mortality even at relatively low volumes, whereas higher pH content was tolerated in larger volumes before mortality was induced.^78^ Acid inhalation studies in rats indicated a 2-stage injury process. Damage occurring within the first hour was attributed to a direct chemical effect on the epithelium; damage ~4 h post-inhalation that coincided with infiltration of the interstitium and alveolar spaces by neutrophils.^85^ In an experimental rabbit model of acid inhalation and acid-induced lung injury,^61^ IL8 was a key chemotactic factor that mediated neutrophil recruitment to the lungs; IL8 neutralizing antibodies protected against clinical signs, mortality, and airspace neutrophil accumulations. These findings support the interpretation that neutrophil influx and interstitial inflammation contribute most of the damage in some experimental models.^61^

Primary acid injury may also facilitate colonization of lung tissue by opportunistic bacteria present in the lower respiratory tract,^113^ complicating the primary sterile event with a secondary bacterial infection. We suggest that the initial acid-related damage is an epithelial ARDS endotype, more likely to induce ARDS when large volumes of very-low pH content are aspirated. In a canine acid-inhalation model, aspiration of a moderate volume of a low pH acid (1 mg/kg, pH 1.8) caused compromise of gas exchange (measured by fractional intrapulmonary physiologic shunt) within 10 min and remained significantly elevated compared with the control for 3 h post injury.^66^ We suggest that damage caused by neutrophil influxes constitutes a local inflammatory ARDS endotype through neutrophil-induced immunopathology on the interstitium.

### Aspiration of bacteria

Aspiration of colonized oropharyngeal or gastric content can result in acute and severe pneumonia. Acute aspiration pneumonia in racing sled dogs (*n* = 8) included ingesta in airways, alveolar edema, fibrin, hemorrhage, neutrophil and macrophage infiltration, and septal necrosis.^44^ Only a proportion of those cases also had bacteria present on histology. A study was designed to determine the prevalence of bacterial involvement in canine aspiration pneumonia; however, the lack of routine bronchoalveolar lavage fluid (**BALF**) collection for cytology and bacterial culture (performed in only 50 of 429 [12%] cases) hampered calculation of the true incidence.^76^ In that study, 19 of the 24 cases meeting the inclusion criteria had bacterial aspiration pneumonia, and 5 had aspiration pneumonia with no bacterial involvement.^76^ A second study had 47 cases of canine aspiration pneumonia that had bacterial culture performed on BALF, and 36 of 47 were positive for bacterial growth.^148^ The species recovered most commonly were *E. coli*, spp., *Pasteurella* spp., and *Staphylococcus* spp.^148^ In 88 cases of canine aspiration pneumonia, survival was not influenced by primary cause, radiographic severity of disease, or length of hospitalization.^88^ This suggests that augmentation of injury by the local inflammatory response is more important in ARDS than the size of the primary injury.

Feline aspiration pneumonia causes acute-onset tachypnea and an alveolar pattern on imaging, with bacterial involvement detected in some cases; however, a lack of routine testing limits the assessment of the true incidence.^41^

### Uremia—endothelial ARDS endotype

In humans, acute kidney injury (**AKI**) has classical pulmonary consequences of volume overload and acidosis; however, uremia also contributes to pulmonary edema through effects on lung endothelial cells.^10,149^ The circulating urea and ammonia causes endothelial apoptosis and lung inflammation.^56^ In a mouse model of AKI, uremia caused increased oxidative stress, ATP depletion, and neutrophil activation in the lungs.^5^ In another mouse model, capillary leak, lung neutrophil infiltrates, and myeloperoxidase activity were increased 4 h after induction of AKI, and the changes were dependent on increased serum IL6.^87^

In humans, lung injury caused by renal failure may progress to ARDS.^100^ In veterinary medicine, uremia is considered a potential cause of ARDS in dogs and cats,^34^ albeit uncommon, especially because cats appear to be relatively resistant to systemic manifestations of uremia.^4^ Based on human and experimental data, uremia is considered an endothelial ARDS endotype, given the effect of circulating factors on the lung endothelium. Lung abnormalities comparable to human uremic pneumonitis were present in 104 dogs with renal azotemia of both acute and chronic causes.^94^ Six of the 104 dogs had respiratory distress or dyspnea, including those with sudden onset of signs; however, clinical detail is not sufficient to retrospectively confirm ARDS. The histologic appearance of uremic lung damage in dogs includes alveolar edema and mineralization of alveolar septa with necrosis of both epithelial and endothelial cells.^94^ In a survey of 78 cats with non-renal lesions of uremia, the most common finding was pulmonary edema (40 of 78 cats). Five of those 40 cats also had uremic pneumopathy with mineralization of alveolar septa, neutrophilic infiltrates, and fibrin deposition. The cats were not in respiratory distress; the most frequent clinical signs were anorexia and apathy. Hyaline membranes were not reported in either the canine or feline study, and therefore these cases did not have concurrent DAD.^4,94^

### Bacterial bronchopneumonia—local inflammatory response ARDS endotype and systemic inflammation ARDS endotype

Bacterial pneumonia is a common cause of ARDS. Hypoxemia and clinical features supportive of vetARDS were common findings in 64 confirmed cases of canine bacterial pneumonia presented to a veterinary teaching hospital.^163^ In dogs and cats, bacterial pneumonia is often precipitated by an event that compromises barrier function, such as an aspiration event, viral infections, inhaled foreign bodies, nosocomial or states of immune dysfunction, or immunosuppression.^40^ From an ARDS endotype perspective, 2 endotypes are potentially applicable: the local inflammatory response endotype, in which local extension of inflammation results in ARDS, and the systemic inflammatory response endotype, in which the primary pneumonia results in sepsis that progresses to sepsis-related ARDS.^11,12^

An understanding of the mechanism by which bacterial bronchopneumonia transitions to ARDS from different primary etiologies is hampered by a lack of prospective controlled studies.^11^

Local inflammation in bacterial bronchopneumonia can be intense and contribute to clinical signs. In single-cell profiling of BALF samples from human patients with bacterial pneumonia, patients with severe bacterial pneumonia, including suspected ARDS cases, experienced a cytokine storm that was not present in patients with mild bacterial pneumonia or healthy controls.^167^ In a mouse model of acute gram-positive pneumonia, lungs had acute severe interstitial pneumonia with extensive neutrophilic infiltrates induced higher levels of IL6, IFNγ, and TNF than those with mild subclinical bacterial bronchopneumonia.^166^ In humans, comparison of cytokine levels between BALF and serum offer evidence of cytokine compartmentalization, indicating that local inflammatory responses in pneumonia could cause ARDS in the absence of sepsis.^12^ For individual veterinary clinical cases, adopting a multidisciplinary approach could help provide evidence to support a specific endotype.

### Pancreatitis—systemic inflammatory response ARDS endotype

Pancreatitis causes respiratory signs that may progress to vetARDS, 33 of 109 client-owned dogs with acute pancreatitis had respiratory signs, including 3 cases of vetARDS^92^; 9 of 26 client-owned dogs with pancreatitis had vetALI.^68^ The American College of Veterinary Internal Medicine consensus statement on feline pancreatitis states that breathing difficulties are common in pancreatitis cases and can be associated with ALI or ARDS.^63^ Clinical cases of canine acute pancreatitis had reduced antithrombin, and elevated TNF, IL6, and C-reactive protein in blood samples, compared with normal controls.^91^ Human cases of pancreatitis also have respiratory complications. In a retrospective study of 359 cases of acute pancreatitis, 31 of 359 (9%) patients developed respiratory failure.^49^ ARDS usually developed 2–7 d after the onset of human pancreatitis and was a major contributor to deaths from acute pancreatitis occurring before hospital admission.^90^

## Conclusion

Causes of ARDS can be conceptually classified by the relative involvement of alveolar, vascular, pulmonary, and systemic inflammation in the primary injury.^19^ The 5 endotypes—epithelial, endothelial, local inflammatory response, angiopathy, and systemic inflammatory response—offer clinically relevant combinations of these attributes. The core premise of ARDS endotypes is that the original injury confers a persistent molecular signature to the ARDS process that differs from those associated with other primary injuries, thereby facilitating precision testing and therapeutics.

In exploring clinical examples of ARDS endotypes applicable to dogs and cats, we highlight that assigning an endotype can be complicated by the multiple potential endotypes and knowledge gaps. We recognize that our designations will be modified in response to future published work, and advances in ARDS understanding and treatment will be iterative. We also believe that work toward defining ARDS endotypes will improve our understanding of the histology of ALI outside of classical DAD, further refining diagnostic accuracy.

We encourage readers to access part 2^165^ of this series, which explores beyond ARDS and DAD to look at other types and causes of NCPE, including the diagnostic challenges presented by pulmonary edema. We also describe downstream immune and cell-death processes involved in ARDS that amplify the primary insult across cellular systems and anatomical locations. In addition, we review the current understanding of comparative immunology of humans, mice, cats, and dogs.^165^
